# Large intraosseous chronic expanding hematoma after total hip arthroplasty presenting with chronic disseminated intravascular coagulation: a case report and literature review

**DOI:** 10.1186/s12891-022-05571-8

**Published:** 2022-06-24

**Authors:** Yuki Yamamuro, Tamon Kabata, Akihiko Takeuchi, Yoshitomo Kajino, Daisuke Inoue, Takaaki Ohmori, Junya Yoshitani, Takuro Ueno, Ken Ueoka, Atsushi Taninaka, Tomoyuki Kataoka, Yoshitomo Saiki, Hiroyuki Tsuchiya

**Affiliations:** Department of Orthopedic Surgery, Kanazawa University, Takaramachi 13–1, Kanazawa, Ishikawa 920-8641 Japan

**Keywords:** Case report, Intraosseous chronic expanding hematoma, Total hip arthroplasty, Chronic disseminated intravascular coagulation, Mosaic sign

## Abstract

**Background:**

A chronic expanding hematoma (CEH) is a rare complication caused by surgery or trauma; it mostly affects the soft tissues, such as those in the trunk or extremities. We present the first case of a large intraosseous CEH presenting with chronic disseminated intravascular coagulation (DIC), 22 years after total hip arthroplasty (THA); the CEH was treated with a single-stage excision and revision THA.

**Case presentation:**

A 67-year-old man presented to our hospital with left thigh pain and an enlarging mass. He had no history of trauma, anticoagulant use, or a collagen vascular disorder. The patient initially declined surgery. Two years later, radiographs and computed tomography images revealed progressive osteolysis, marginal sclerosis, and calcification in the left femur, in addition to loosening of the femoral component. Laboratory data revealed anemia and chronic DIC of unknown causes. Magnetic resonance imaging revealed a “mosaic sign” on the mass, indicating a mix of low- and high-signal intensities on T2-weighted images. Needle biopsy prior to surgery revealed no infection or malignant findings. An intraosseous CEH was suspected due to extensive osteolysis and loosening of the femoral component. No other factors that could induce chronic DIC were identified, such as sepsis, leukemia, cancer, trauma, liver disease, aneurysms, or hemangiomas. Therefore, we speculated that the anemia and chronic DIC were caused by the large intraosseous CEH. A single-stage revision THA with surgical excision was performed to preserve the hip function and improve the chronic DIC. The postoperative histopathological findings were consistent with an intraosseous CEH. The anemia and chronic DIC improved after 7 days. There was no recurrence of intraosseous CEH or chronic DIC at the 6-month follow-up. The left thigh pain improved, and the patient could ambulate with the assistance of a walking frame.

**Conclusions:**

The loosening of the femoral component caused persistent movement, which may have caused intraosseous CEH growth, anemia, and chronic DIC. It is important to differentiate CEHs from malignant tumors with hematomas. Furthermore, the “mosaic sign” noted in this case has also been observed on magnetic resonance images in other cases of CEH.

## Background

Chronic expanding hematoma (CEH) is a rare complication resulting from trauma or surgery and can develop at various locations (including the trunk or soft tissue of the extremities) over the course of a month after the initial bleeding [1]. It shows no neoplastic changes on histological sections, and does not occur in the setting of coagulopathy [1]. CEHs, including intraosseous CEHs (an extremely rare complication), involve the risk of progression to anemia and occasionally require surgical treatment. However, a large intraosseous CEH presenting with chronic disseminated intravascular coagulation (DIC) has not been reported previously. Herein, we present the case of a patient who developed a large intraosseous CEH with chronic DIC 22 years after total hip arthroplasty (THA); the patient was treated with a single-stage excision and revision THA using a tumor prosthesis.

## Case presentation

**Fig. 1:**
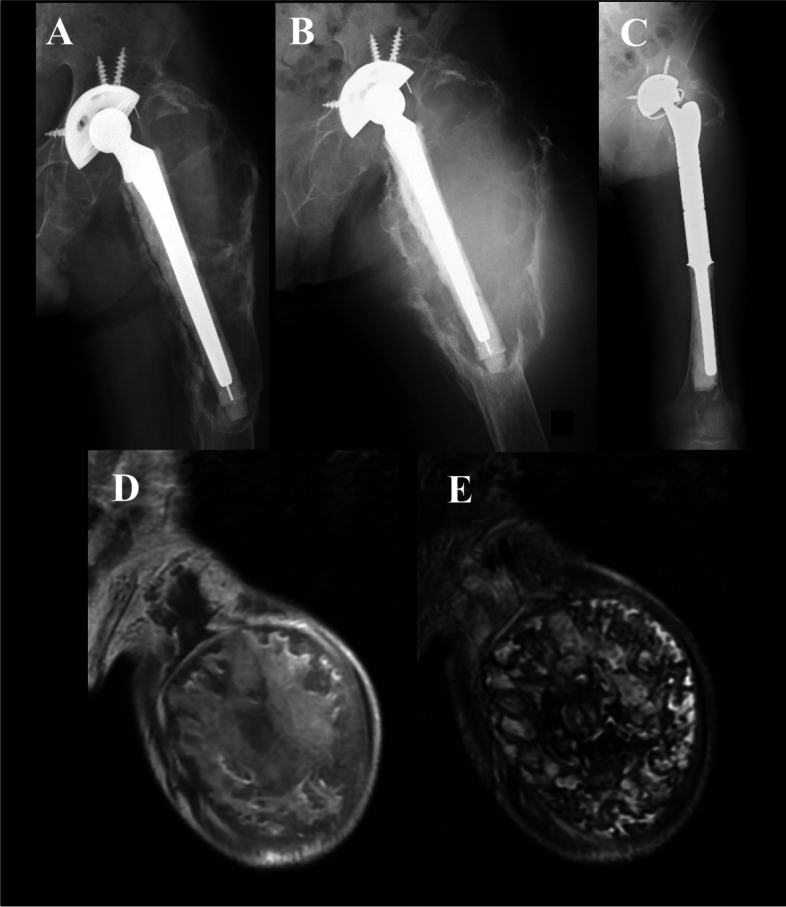
**(A)** Initial radiograph taken at the first presentation reveals osteolysis, marginal sclerosis, and calcification in the femur, as well as the loosening of the femoral component. **(B)** Preoperative radiograph taken 2 years after the first visit reveals progressive osteolysis, marginal sclerosis, and calcification in the left femur. **(C)** No recurrence of intraosseous chronic expanding hematoma is observed at the most recent follow-up, i.e., 6 months postoperatively. The mass predominantly shows a low-signal intensity on a T1-weighted magnetic resonance image **(D)** and a mixture of low- and high-signal intensities (“mosaic sign”) on a T2-weighted magnetic resonance image **(E)**

A 67-year-old man presented to our hospital with left thigh pain and an enlarging mass. His height and weight at the first visit were 160 cm and 51.5 kg, respectively (body mass index, 20.1 kg/m^2^). He had a history of ankylosing spondylitis and chronic renal failure. He had undergone left THA for ankylosing spondylitis when he was 45 years old. There was no history of trauma, anticoagulant use, or collagen vascular disorder. At the first visit, an initial radiograph revealed osteolysis, marginal sclerosis, calcification in the femur, and loosening of the femoral component (Fig. 1A). There were no apparent signs of anemia or chronic DIC. Needle biopsy revealed granulation tissue with lymphocyte infiltration as well as fibrocyte infiltration with foam cells and some spindle-shaped nuclei; no infection or malignant findings were noted. Revision THA was considered necessary and was recommended to the patient due to osteolysis and loosening of the femoral component; however, the patient initially declined as he was able to ambulate with a walking frame and did not wish to undergo surgery. At 2 years after the first visit, the left thigh pain worsened, the mass increased in size, and he was unable to walk. Physical examination revealed a firm, immobile mass in the left thigh. There were no neurological signs of motor or sensory disturbances in the limbs, and no inguinal lymph-node swelling was observed. The patient was anemic (hemoglobin 8.7 g/dL) with a platelet count of 50 × 10^3^/μL and the International Society on Thrombosis and Hemostasis (ISTH) DIC score of 5 points [2].

Preoperative radiographs and computed tomography images revealed progressive osteolysis, marginal sclerosis, and calcification (Fig. 1B). Contrast-enhanced computed tomography revealed feeding arteries from the deep femoral artery to the mass. T1-weighted magnetic resonance imaging (MRI) revealed that the mass had a predominantly low signal intensity, while T2-weighted MRI revealed a mixture of low and high signal intensities that formed a “mosaic sign” (Fig. 1D and E). A large intraosseous CEH was suspected that occurred 22 years after THA. No other factors that could have induced the chronic DIC were identified (e.g., sepsis, leukemia, cancer, trauma, liver disease, aneurysm, or hemangioma). Therefore, the anemia and chronic DIC were hypothesized to have been caused by a large intraosseous CEH. A single-stage revision THA with surgical excision was performed with surgical excision to treat the patient’s symptoms, preserve the hip function, and improve the chronic DIC.

### Surgical management and follow-up

**Fig. 2:**
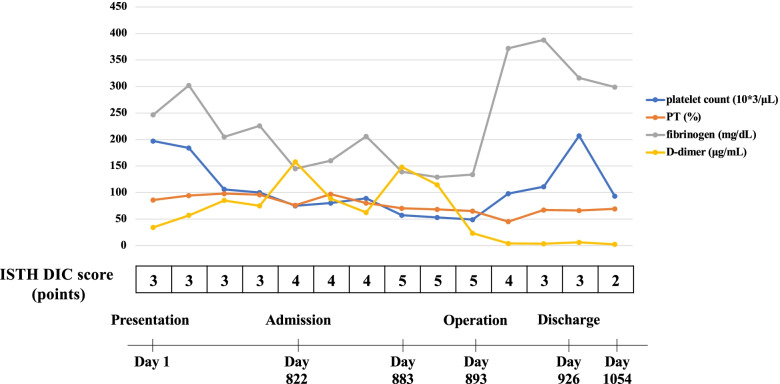
Clinical course from the presentation to the final follow-up. The changes in the coagulo-fibrinolytic parameters are presented in the diagrams. The status of disseminated intravascular coagulation improved after the surgery. ISTH DIC, International Society on Thrombosis and Haemostasis disseminated intravascular coagulation; PT, prothrombin time

We planned a single-stage excision and revision THA after obtaining signed informed consent. The Hueter’s anterior approach was chosen, because the large intraosseous CEH was in contact with the neurovascular bundle and required an adequate release. Macroscopic examination revealed that the lesion was encased within the cortical bone. The deep femoral artery was identified, and a branch of the deep femoral artery that supplied the mass was ligated. The authors of a previous case report on an intraosseous CEH identified the feeding vessels similarly [1]. Frozen sections revealed no evidence of malignancy. Osteotomy was performed just distal to the stem, and the greater trochanter was preserved. The mass was excised with the stem and a portion of the adherent surrounding muscle. No gross polyethylene wear was observed. The acetabular cup was stable and in a good position; thus, it was preserved. Revision THA was performed using a tumor prosthesis (surgery duration, 5 h 57 min); a constrained acetabular liner was used. The total procedural blood loss, including blood clots from the hematoma, was 3070 mL. Intraoperatively, the patient received a transfusion of 22 units of packed red cells; preoperatively, the patient received a 100 mL platelet transfusion for the chronic DIC. Furthermore, to treat the chronic DIC, nafamostat mesylate (200 mg/day) was infused continuously. The fascia was carefully repaired, and drains were placed. Postoperative drains were retained until 6 days postoperatively. The ISTH DIC score was 4 points on day 3 and improved to 3 points on day 7 (Fig. 2). The patient’s postoperative course was uneventful. There was no recurrence of the intraosseous CEH based on physical, radiographic, and computed tomography examinations performed at the most recent follow-up (i.e., at 6 months postoperatively; Fig. 1C). Furthermore, there was also no recurrence of anemia or chronic DIC. The left thigh pain improved, and the patient was able to ambulate with the assistance of a walking frame.

### Histopathologic findings

**Fig. 3:**
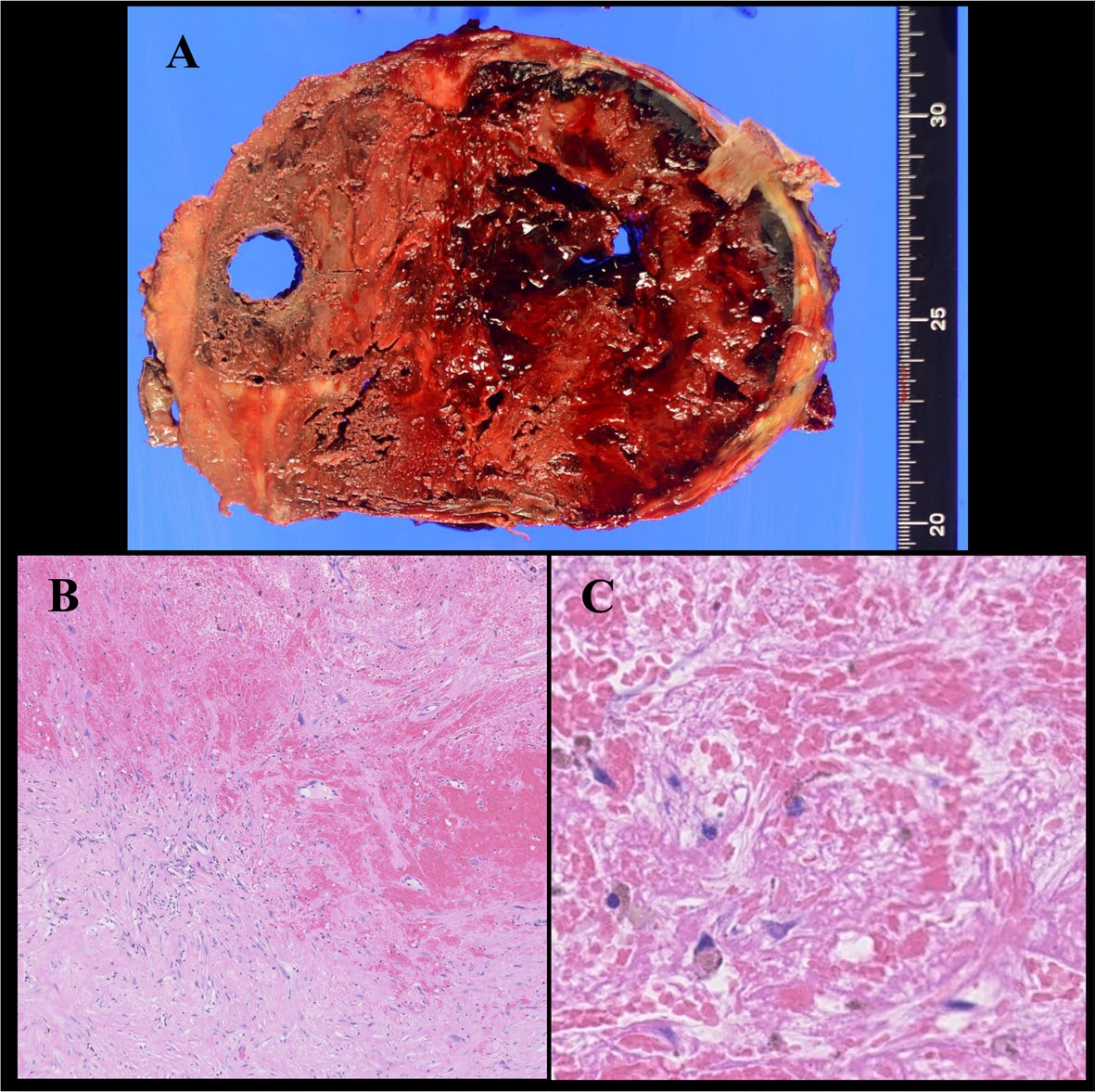
**(A)** The lesion is noted to be 20 × 17 × 12 cm in size; it weighed 2350 g. The mass is seen to contain rust-colored old blood and a large amount of fibrinous tissue encased within the cortical bone. **(B, C)** Histopathological examination reveals a multitude of old blood clots and fibrous tissue membranes lined by foamy and hemosiderin-laden macrophages. No evidence of neoplastic tissue with hemorrhage is seen, and the mass is noted to be composed of blood clots and fibrin exudation. Magnification: **B**, × 5; **C**, × 40

The lesion was 20 × 17 × 12 cm in size and weighed 2350 g. The mass contained rust-colored old blood and a large amount of fibrinous tissue encased within the cortical bone (Fig. 3A). Histopathologic examination revealed many old blood clots and fibrous tissue membranes with a lining of foamy and hemosiderin-laden macrophages (Fig. 3B and C). Therefore, the final diagnosis was of a post-THA femoral intraosseous CEH.

## Discussion and conclusions

This is the first reported case of a large intraosseous CEH that presented with chronic DIC and occurred 22 years after THA; it was successfully treated with a single-stage excision and revision THA. In 2018, Morishita et al. [3] first reported a case of a rare complication wherein a CEH arose from the iliac bone following a hip surgery. To the best of our knowledge, this is the only other reported case of an intraosseous CEH. In the present case, there was no history of anticoagulant use or a collagen vascular disorder. Moreover, the hematoma arose from the proximal femur and expanded gradually; it showed no evidence of neoplastic tissue during histopathological examination.

The large intraosseous CEH was the cause of anemia and chronic DIC in our patient. Previous studies have shown that CEH causes anemia [4, 5]. Nevertheless, no case of abnormal coagulation due to a CEH has been reported. Chronic DIC is a life-threatening process that can lead to thrombosis and hemorrhage. Some patients with chronic DIC develop hemorrhagic diathesis, which is first detected when trauma or surgery triggers a difficulty in achieving hemostasis. In the absence of such triggers, the patients may be asymptomatic; chronic DIC may go undetected despite coagulation-fibrinolytic activation, partly due to the compensatory mechanisms that lead to mild or even occult clinical symptoms. Chronic DIC is an acquired disorder that occurs in patients with various clinical conditions, including carcinomatosis, retained dead fetus, liver disease, aneurysms, and hemangiomas [6]. In particular, a previous study on aortic aneurysms indicated that DIC is mainly related to giant aortic aneurysms with a large diameter (> 7 cm), especially expanding aneurysms [7]. It is possible that, in a similar fashion to DIC generation in aortic aneurysms, local plasmin and thrombin generation in a large intraosseous CEH is so great that the platelets and coagulation factors are depleted, leading to chronic DIC. The following criteria are used for establishing aortic aneurysms as the primary etiology of DIC: presence of a chronic acquired bleeding disorder, laboratory evidence of DIC, correction of hemostatic abnormalities by successful repair, and maintenance of normal coagulation for at least 3 months thereafter [7]. Based on these criteria and after excluding other factors that can induce chronic DIC, we concluded that a large intraosseous CEH led to the chronic DIC in the present case. It is important to exclude anemia and chronic DIC in patients with large intraosseous CEHs.

It is important to differentiate between CEHs and malignant tumors with hematomas, because a CEH may be misdiagnosed as a malignant tumor. The mass location, size, and calcification or spatial resolution on radiography and CT are useful for establishing a diagnosis. However, both new and old hematomas show almost perfectly uniform densities; hence, distinguishing between them is reportedly difficult [8]. MRI is the gold standard modality for differentiating a malignant tumor from a hematoma. Akata et al. described a “mosaic sign” on T2-weighted images, which comprised multiple internal signal intensities secondary to the degrading blood products of various ages [9]. A CEH appears as a heterogeneous signal pattern, which reflects a collection of fresh and altered blood throughout the lesion. MRI is a good diagnostic modality for CEHs, which often show a mixture of low- and high-intensity areas on T1- and T2-weighted images; however, it is difficult to distinguish these lesions from malignancies using MRI. Furthermore, in a previously reported case, a mass that was diagnosed as a hematoma on biopsy was eventually identified as a malignant tumor [10]. Therefore, in the diagnosis of CEH, it is important to know the patient’s history of trauma or surgery and thoroughly assess the MRI findings and clinical information to distinguish a CEH from a malignant tumor [8, 10]. In the present case of an intraosseous CEH, the 22-year history of THA and the “mosaic sign” on T2-weighted images helped confirm the diagnosis. Moreover, pathological findings of the resected tumor were consistent with those of an intraosseous CEH.

In the present case, anemia and chronic DIC were observed with the growth of the mass. A critical point in the management of chronic DIC is the eradication of the primary disease and treatment of concomitant causes. Accordingly, surgical excision was performed to improve the anemia and chronic DIC. A tumor prosthesis made it possible to reconstruct the bone defects and preserve the joint function. No recurrence was noted at 6 months postoperatively. A limitation of this study was the relatively short follow-up period. Yet, we were able to improve the left thigh pain, anemia, and chronic DIC and maintain the patient’s hip function with a single-stage excision and revision THA. To date, the patient has presented a satisfactory course without recurrence; however, he has been advised to undergo long-term follow-up for monitoring a recurrence. In conclusion, in case of a suspected large intraosseous CEH, chronic DIC should be ruled out. The persistent movements caused by the loosening of the femoral component may have caused intraosseous CEH growth, anemia, and even chronic DIC.

## Data Availability

The datasets used and/or analyzed during the current study are available from the corresponding author on reasonable request.

## Abbreviations

CEH: chronic expanding hematoma
DIC: disseminated intravascular coagulation
ISTH: International Society on Thrombosis and Haemostasis
MRI: magnetic resonance imaging
PT: prothrombin time
THA: total hip arthroplasty
